# GuiLingJi ameliorates mild cognitive impairment by targeting unsaturated fatty acid metabolism to inhibit GPR120/NF-κB mediated neuroinflammation

**DOI:** 10.3389/fphar.2026.1729885

**Published:** 2026-03-11

**Authors:** Jingchao Shi, Lingfan Ni, ShuTing Yu, Xiaoxia Gao, Xuemei Qin

**Affiliations:** 1 School of Traditional Chinese Materia Medica and Food Engineering, Shanxi University of Chinese Medicine, Jinzhong, China; 2 Taihang Institute of Materia Medica, Shanxi University of Chinese Medicine, Jinzhong, China; 3 Modern Research Center for Traditional Chinese Medicine, Shanxi University, Taiyuan, Shanxi, China

**Keywords:** Guilingji, metabolomics, mild cognitive impairment, neuroinflammation, unsaturated fatty acids metabolism

## Abstract

**Background:**

Mild cognitive impairment (MCI) is an intermediate condition between normal aging and dementia. Drug intervention is an important way to prevent MCI from developing into dementia. GuiLingJi (GLJ) is a traditional Chinese medicine formulae and it has the effect of enhancing memory. In view of the absence of special effective drugs for MCI, GLJ warrants investigation as a potential therapeutic agent.

**Methods:**

This study uses a rat model of MCI, induced by D-galactose injections and a semi-high-fat diet, to explore the therapeutic efficacy of GLJ in MCI and elucidate the potential underlying pharmacological mechanisms by behavioral experiments and biochemical indexes, combined with serum and hippocampal metabolomics.

**Results:**

GLJ treatment mitigated D-galactose combined with semi-high-fat diet induced impairments, including abnormal blood lipids, oxidative stress, inflammation, cholinergic dysfunction, apoptosis, and reduced brain-derived neurotrophic factors, along with hippocampal damage. LC-MS metabolomics indicated that these effects involved unsaturated fatty acid and amino acid metabolism. By normalizing linoleic acid and α-linolenic acid levels and activating GPR120, GLJ inhibited the NF-κB/TNF-α pathway.

**Conclusion:**

These findings demonstrate that GLJ alleviates MCI symptoms, at least in part, by modulating fatty acid metabolism and suppressing neuroinflammation *via* the GPR120/NF-κB pathway. This study supports GLJ as a promising proprietary TCM formulation for MCI treatment.

## 1 Introduction

The rising global elderly population has significantly increased public health concerns regarding cognitive disorders such as mild cognitive impairment (MCI) and dementia. MCI represents a phase of cognitive decline that lies somewhere between the typical age-related cognitive decline and the early stages of mild Alzheimer’s disease (AD). Individuals with MCI exhibit perceptible cognitive deficits, including memory impairment, while maintaining their ability to perform daily activities (Chen et al., 2023). On average, the rate of progression of MCI to AD is significantly higher at 10%–15%, compared to the 1%–2% progression rate observed in individuals with normal aging, which makes MCI a key area of interest in clinical research (Kumar et al., 2023). Approximately 21% of elderly individuals with normal cognitive function eventually develop MCI, and they are 2.8 times more likely to advance towards dementia compared to those with average cognition (Harris et al., 2023). Due to the lack of effective disease-modifying therapies for advanced dementia, MCI represents a critical stage at which interventions aimed at delaying or preventing the transition to dementia can be implemented (Werneck et al., 2023). There is insufficient conclusive evidence regarding the therapeutic efficacy of current pharmacological interventions, such as donepezil, for MCI. Aducanumab is the first FDA-approved drug for the treatment of MCI and early AD, and lecanemab can reduce the levels of beta-amyloid (Aβ) plaques. However, both aducanumab and lecanemab need further research is necessary for confirming their safety and efficacy (van Dyck et al., 2023). Therefore, the exploration of effective drugs for the treatment of MCI from the extensive resources of traditional Chinese medicine (TCM) holds significant potential.

GuiLingJi (GLJ) is an effective Chinese herbal formulation and has been used for over 400 years. GLJ has the efficacy of treating Kidney-Yang deficiency syndrome, enhancing brain function, and improving memory. The composition of GLJ was listed in Supplementary Table S1, and all the plant names has been checked with “The Plant List” (www.theplantlist.org) or MPNS (http://mpns.kew.org). The rest of animal and mineral herbs are listed by their Latin names. Studies revealed that the metabolites of GLJ, including flavonoids, ginsenosides, coumarins, and phenolic acids (Shi et al., 2022; Zhang et al., 2023; Zhang et al., 2021), exhibit diverse pharmacological activities such as anti-inflammatory effects, neuroprotection, cognitive improvement, antifatigue properties, and reproductive function enhancement (Mishra et al., 2020; Niu et al., 2020; Yang et al., 2023). Pharmacological studies suggest that GLJ exhibits therapeutic potential in treating reproductive dysfunction across multiple animal models, including improving spermatogenesis in oligoasthenoteratozoospermia rats, Immp2l mutant mice and sexual dysfunction rats by regulating lipid/amino acid metabolism, antioxidant balance, and mitogen-activated protein kinase signaling pathways, with arachidonic acid metabolism (Ding et al., 2022; Wang et al., 2022; Zhu et al., 2024). GLJ can also significantly improve cognitive dysfunction in rats with hydrocortisone- and Aβ-induced AD by protecting the hippocampal tissue, regulating the serum levels of cortisol and testosterone, and correcting the metabolic disorders in primary bile acid and phospholipid biosynthesis (Ren et al., 2022). Using several animal models of aging, we have previously Using several animal models of aging, we have previously demonstrated that GLJ targets oxidative stress, hormonal imbalance, and metabolic dysfunction to ameliorate memory decline in aged animals (Yang et al., 2020; Zhao et al., 2020; Zhao et al., 2019; Zhao et al., 2018). Clinical research had also shown that GLJ can effectively enhance cognitive functions, especially for individuals experiencing mild to moderate Alzheimer’s Disease or vascular mild cognitive impairment (v-MCI), without causing any severe adverse events during trials (Liu et al., 2020; Zhang et al., 2022; Zhao et al., 2023). This study builds upon previous research to investigate GLJ’s therapeutic effects and underlying mechanisms in a rat model of MCI, aiming to identify a more effective early intervention strategy for age-related cognitive decline.

## 2 Materials and methods

### 2.1 Medicine and reagents

GLJ (Approval Number: Z1402068) was procured from Shanxi Guangyuyuan Chinese Medicine Co., Ltd. (Shanxi, China). The quality of the GLJ used in this study was assessed and confirmed through three orthogonal fingerprinting analyses, with the representative chromatograms provided in Supplementary Figures S1-S3. Its metabolites have already identified (Shi et al., 2022), and the total ion chromatograms of GLJ were shown in Supplementary Figure S4. Ginkgo biloba tablets (Approval Number: Z20027949), purchased from Yangtze River Pharmaceutical Co., Ltd. (Jiangsu, China), and donepezil hydrochloride (Lot No. 1705045), purchased from Eisai China Inc. (Shanghai, China) were used as the positive control drugs. D-galactose (D-gal, Lot No. 102030630) was procured from Sigma-Aldrich Trading Co., Ltd. (Shanghai, China). The semi-high-fat feed (5% lard +0.5% cholesterol +5% egg yolk powder +89.5% basal feed) was purchased from Beijing Keao Xieli Feed Co., Ltd. (Beijing, China). Ultra-pure water was prepared using a MilliQ Integral Water Purification System (Massachusetts, United States). Acetonitrile (LC-MS grade) and formic acid (HPLC grade) were purchased from Thermo Fisher Scientific Inc. (Waltham, United States).

### 2.2 Animals and treatments

Totally 60 male Sprague-Dawley (SD) rats, weighing 180 ± 20 g, were obtained from the Experimental Animal Center of Beijing Weitonglihua Technology Co., Ltd. (license number: SCXK (JING) 2019–0006). The animals were kept under typical lab settings, with a consistent 12-h alternating light and dark schedule, at an ambient temperature of 24 °C ± 1 °C and a relative humidity level of 50% ± 5%. Before the experiments began, the animals had a week-long adjustment period to adapt to these lab conditions. The 60 rats were assigned to six distinct groups in a random manner, taking their body weights into consideration (n = 10): control group (Ctrl), model group (Model), GLJ group that received 150 mg/kg/day GLJ (High GLJ), GLJ group that received 75 mg/kg/day GLJ (Low GLJ), a positive control group that received Ginkgo biloba tablets at a dose of 7.2 mg/kg/day (EGB), and a positive control group that received donepezil hydrochloride at a dose of 0.625 mg/kg/day (Donepezil). The equivalent rat dosages were calculated based on the original prescription recorded in GLJ, Ginkgo biloba tablets and Donepezil, and body surface area conversion (coefficient 6.3).

The Model group received a chronic injection of D-gal (150 mg/kg/day) in combination with a semi-high-fat diet throughout the experimental period for 8 weeks. The treatment methods for each administration group were the same as those of the model group. And the Ctrl group of rats were given an equivalent volume of physiological saline through injection. The High GLJ, Low GLJ, EGB and Donepezil groups intragastric administration the suspensions of GLJ, EGB, and Donepezil prepared by 0.5% sodium carboxyl methyl cellulose (CMC-Na) respectively. Ctrl and Model groups received a daily gavage of an equivalent volume of 0.5% CMC-Na suspension. The experimental protocol was approved by the Animal Ethics Committee of Shanxi University (approval number: 2020DW121).

All experiments were performed in accordance with ARRIVE guidelines.

### 2.3 Morris water maze test

Cognitive functions, including spatial learning and memorization ability, were evaluated using the Morris water maze test on the final day of the 8th week. The test was conducted in a circular pool (diameter: 180 cm; height: 60 cm) filled with opaque water (maintained at 22 °C ± 1 °C). A hidden escape platform (diameter: 12 cm) was submerged 1.5 cm below the water surface in a fixed quadrant. Acquisition Training: Over four consecutive days, each rat underwent four trials per day from different starting points. Rats were allowed to search for the platform for a maximum of 60 s. Upon finding it, they remained there for 5 s. However, the rats that failed to locate the platform within 60 s were manually placed on the platform for 20 s. Probe Trial: On the fifth day, the platform was removed. Each rat performed a single 60-s free swim. Time spent in the target quadrant and the number of platform crossings were analyzed as indices of memory retention (Muhammad et al., 2022).

### 2.4 Sample collection

Upon completion of the MWM probe trial, rats were deeply anesthetized. Blood was collected *via* cardiac puncture into sterile tubes. Subsequently, animals were perfused transcardially with ice-cold phosphate-buffered saline. The brain was rapidly extracted, and the bilateral hippocampi were carefully dissected on an ice-cold plate. Blood samples were kept at 4 °C for 30 min to clot, then centrifuged at 3,500 × g for 15 min at 4 °C. The supernatant was aliquoted and stored at −80 °C until biochemical and metabolomic analysis.

One hemisphere of the hippocampus was immersed (i.e., fully submerged) in a 10-fold volume excess of 4% paraformaldehyde (PFA) in 0.1 M phosphate buffer (pH 7.4) at 4 °C for 24 h for fixation. After fixation, tissues were transferred to a 30% sucrose solution for cryoprotection. The contralateral hippocampal hemisphere was immediately flash-frozen in liquid nitrogen and stored at −80 °C for subsequent biochemical and metabolomic analysis.

### 2.5 Serum biochemical analysis

Commercial assay kits were used for biochemical analyses. Serum lipid profiles, including total cholesterol (TC; Cat# AD3319Ra), triglycerides (TGs; Cat# AD3320Ra), high-density lipoprotein cholesterol (HDL-C; Cat# AD3321Ra), and low-density lipoprotein cholesterol (LDL-C; Cat# AD3322Ra) were determined to use enzymatic colorimetric kits. Oxidative stress markers were assessed as follows: malondialdehyde (MDA; Cat# AD2870Ra) was measured by a competitive enzyme-linked immunosorbent assay (ELISA), while activities of superoxide dismutase (SOD; Cat# AD2871Ra) and glutathione peroxidase (GSH-Px; Cat# AD3318Ra) were determined using enzymatic colorimetric kits. All the above kits were obtained from Beijing Andy Huatai Technology Co., Ltd. (Beijing, China). The serum levels of inflammatory cytokines include tumor necrosis factor-α (TNF-α; Cat# F16960), interleukin-1β (IL-1β; Cat# F15810) and interleukin-6 (IL-6; Cat# F15870) were quantified using specific ELISA kits purchased from Shanghai Westang Bio-tech Co., Ltd. (Shanghai, China).

### 2.6 Biochemical analysis and histopathology of hippocampal tissue

Commercial kits from Shanghai Westang Bio-tech Co., Ltd. (Shanghai, China) were used to assess hippocampal biomarkers. The levels of acetylcholine (ACh; Cat# F15023) and brain-derived neurotrophic factor (BDNF; Cat# F15100) were quantified using ELISA kits. Similarly, the protein levels of apoptosis regulators B-cell lymphoma-2 (Bcl-2; Cat# F15155) and Bcl-2-associated X protein (Bax; Cat# F15154) were also measured by ELISA. In addition, the activity of acetylcholinesterase (AChE; Cat# G0016) was determined using an enzymatic colorimetric assay kit.

Hippocampal tissues were fixed (48 h), paraffin-embedded, sectioned (4 mm thick), and stained with hematoxylin and eosin (HE). The histological sections were scrutinized using a light microscope (×400), and the images were subsequently acquired. Quantitative analysis of pyramidal cell in hippocampal CA1 was using ImageJ software (National Institutes of Health, Bethesda, MD, United States, version 1.53). 2.7 Preparation of metabolomics samples.

The serum samples were prepared by protein precipitation, by adding 300 μL of a 0.1% solution of acetonitrile to 100 μL of the serum samples. After vortexing the solution for 2 min, it was centrifuged at 13,000 rpm for 15 min at 4 °C. The supernatant liquid was then carefully removed and evaporated to dryness. The residue was further dissolved in 100 μL of a 0.1% solution of acetonitrile, centrifuged at 13,000 rpm for 15 min at 4 °C, and the resulting supernatant was collected for further analysis. Serum samples of 10 μL from each group were mixed and prepared as QC samples to measure system stability.

The hippocampal tissues were thawed out at 4 °C, and subsequently weighed and homogenized with 0.2% acetonitrile for 2 min. The homogenate was centrifuged at 13,000 rpm for 15 min at 4 °C, and the resulting supernatant was collected and evaporated to dryness. The residue was dissolved with 50 μL of a 0.1% formic acid water - acetonitrile (9:1), and the mixture was centrifuged at 13,000 rpm for 15 min at 4 °C. The resulting supernatant is then collected for further analysis.

#### 2.6.1 Metabolomics analysis

In this study, liquid chromatography (LC) was performed using a Nexera LC-40 UPLC system (Shimadzu Corporation, Japan), equipped with an autosampler and a column oven for temperature control. A Waters ACQUITY UPLC HSS T3 column (2.1 mm × 100 mm, 1.7 μm; Waters Corporation, Milford, United States) was used for the analysis. The HPLC method was developed in accordance with references from the literature (Du et al., 2020).

For both the serum and hippocampal samples, A TripleTOF 5,600+ system was used for Mass spectrometry (MS) analysis (AB SCIEX Corporation, Singapore City, Singapore). SWATH mode was used for data collection, mass range: 80∼1,200 m/z, production scan: 50∼1,500 m/z, ion spray voltage: 5.5 kV/-4.5 kV, and declustering potential: 60/-60 V. The collision energy was 45 eV/-50 eV.

#### 2.6.2 Data processing and analysis of multivariate patterns

The raw flies obtained by LC-MS were converted using the One-MAP/PTO software, version 2.0 (Dalian ChemDataSolution, Dalian, China). The analysis parameter: mass tolerance: 5 ppm, min peak width/max peak width: 5–15, method: obiwarp, and signal-to-noise threshold: 5. A maximum of 30 fragments were retained for extracting the secondary mass spectrometry data.

The SIMCA-P software (version 14.1, Umetrics, Sweden) was used to conduct principal component analysis (PCA), partial least-squares discriminant analysis (PLS-DA), and orthogonal partial least-squares discriminant analysis (OPLS-DA). The variable importance in projection (VIP) score of variables in projection indicates the significance of different variables. The differential metabolites were screened with VIP >1, *p* < 0.05, and P-corr >0.58. The metabolites were initially identified using the OSI/SMMS software for the rapid analysis of small molecule metabolites (Dalian ChemDataSolution, Dalian, China), based on MS2 data.

The possible structures of the MS/MS fragments identified by the software were compared to the HMDB (http://www.hmdb.ca/spectra/ms/search), mzcloudTM (https://www.mzcloud.org), Lipid Maps (http://www.lipidmaps.org), MassBank (http://www.massbank.jp), and Metlin (http://metlin.scripps.edu) databases. The structures of the differential metabolites were identified by the MS/MS data and their chromatographic retention behaviors.

The potential significance of the small molecule metabolites and biochemical indices was assessed by correlation analysis between the biomarkers and the serum and hippocampal biochemical indices. The differential metabolite peak area data and biochemical indices, including the lipid levels, oxidative stress markers, and levels of inflammatory factors in the serum, as well as the cholinergic, neurotrophic, and apoptotic indices in the hippocampus, were standardized and processed by Spearman correlation analysis using the IBM SPSS software, version 25.0 (SPSS Inc. Chicago, IL, United States). A correlation heat map was finally generated using the Wu Kong platform (https://www.omicsolution.com/wkomics/main/).

Further analyses were performed using the MetaboAnalyst software, version 6.0 (https://www.metaboanalyst.ca/faces/home.xhtml), and the Kyoto Encyclopedia of Genes and Genomes database (KEGG, http://www.genome.jp/kegg/), to obtain further information regarding the metabolic pathways.

### 2.7 Western blot analysis

Hippocampus tissue proteins were extracted using RIPA buffer and quantified using both the BCA and Bradford methods. Following separation by SDS-PAGE and transfer to a PVDF membrane, the blots were incubated overnight at 4 °C with primary antibodies against GPR120 (1:750), NF-κB (1:750), TNF-α (1:350), and GAPDH (1:3,000). Subsequently, the membranes were incubated with an HRP-conjugated goat anti-rabbit secondary antibody (1:15,000) for 2 h. Protein bands were visualized *via* enhanced chemiluminescence (ECL), and densitometric analysis was performed using ImageJ software, with GAPDH serving as the loading control. GPR120 antibody (Cat# A18689) was purchased from ABclonal Biotechnology Co., Ltd. (Wuhan, China). NF-κB (Cat# GB15997), TNF-α (Cat# GB155702), GAPDH (Cat# GB15004), pertained protein marker (Cat# G2087), 5×SDS-PAGE protein loading buffer (Lot No. G2075) and secondary antibodies (Cat# GB23303) were purchased from Wuhan Servicebio Technology Co., Ltd. (Wuhan, China).

### 2.8 Statistical analysis

Statistical analyses were performed using the IBM SPSS software, version 25.0 (SPSS Inc. Chicago, IL, United States). The Morris water maze escape latency data were using two-way repeated-measures ANOVA, followed by Tukey’s *post hoc* test for specific day comparisons where appropriate. For other types of data, comparisons among multiple groups were performed by one-way analysis of variance (ANOVA) followed by Tukey’s honest significant difference (HSD) *post hoc* test for pairwise comparisons if the ANOVA result was significant (*p* < 0.05). All data are reported as mean ± standard error of the mean (SEM).

## 3 Results

### 3.1 Effects of GLJ on the rat model of MCI in behavioural

**FIGURE 1:**
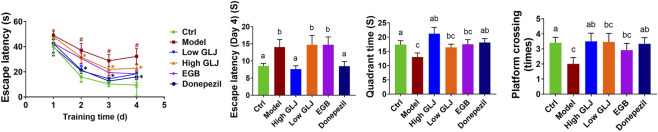
Results of Morris water maze experiments in each group of rats. Statistical significance of escape latency was determined by two-way repeated-measures ANOVA followed by Tukey’s *post hoc* test, the others were determined by one-way ANOVA followed by Tukey’s *post hoc* test, #*p* < 0.05 vs. Ctrl group; *p < 0.05 vs. Model group. Letters a, b, c indicate groups with *p* < 0.05 by Tukey’s test.

The results of the Morris water maze test revealed that, During the four-day training period, all groups showed a progressive decrease in escape latency (Figure 1). Two-way repeated-measures ANOVA revealed a significant main effect of Day (*p* < 0.05) and a significant Group × Day interaction (*p* < 0.05). On the final training day (Day 4), the Model group exhibited significantly longer latencies compared to the Ctrl group (*p* < 0.05). In contrast, both high-dose GLJ and donepezil significantly shortened the escape latency compared to the Model group (*p* < 0.05). Similar trends were observed in platform crossings and target quadrant duration, with Model rats displaying significantly fewer crossings and less time in the target quadrant than controls (*p* < 0.05). The High GLJ and Donepezil groups demonstrated significantly increased platform quadrant residence time (*p* < 0.01 and *p* < 0.05, respectively) and crossing frequency (*p* < 0.05). No significant differences were found between the EGB and Model groups in these measures (Figure 1).

### 3.2 Effects of GLJ on the rat model of MCI in serum biochemical indicators

**FIGURE 2:**
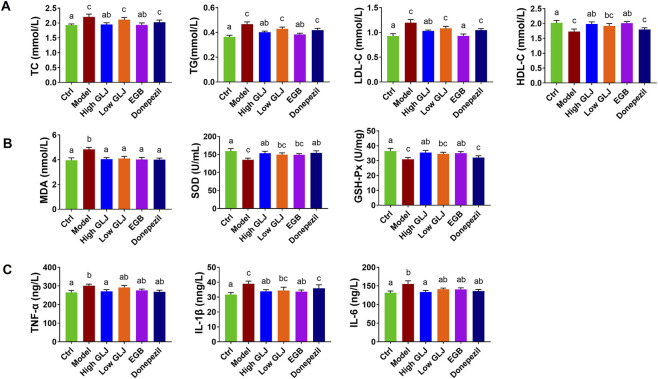
Effects of GLJ on the rat model of MCI in serum biochemical indicators. **(A)** The serum lipid levels of each group of rats. **(B)** The serum levels of oxidative stress of each group of rats. **(C)** The serum levels of inflammation factors of each group of rats. Statistical significance was determined by one-way ANOVA followed by Tukey’s *post hoc* test. Letters a, b, c indicates groups with *p* < 0.05 by Tukey’s test.

The Model group exhibited significantly elevated serum TC, TG, and LDL-C levels (*p* < 0.05) and reduced HDL-C (*p* < 0.05) compared to the Ctrl group. Treatment with High GLJ and EGB significantly lowered TC, TG, and LDL-C (High GLJ: *p* < 0.05 for all; EGB: *p* < 0.05, *p* < 0.01, and *p* < 0.05, respectively) and increased HDL-C in the EGB group (*p* < 0.05). In contrast, Low GLJ and Donepezil groups showed non-significant trends toward normalization. These results indicate that high dose GLJ and EGB can effectively modulate the blood lipid levels of rats with hyperlipidemia caused by D-gal combined with semi-high-fat diet (Figure 2A).

The oxidative stress indicator indicated a significant increase in MDA levels in the Model group, whereas the activity of SOD and GSH-Px showed a significant decrease compared to the Ctrl group (*p* < 0.05 for all; Figure 2B). These results indicated that the rat model of MCI exhibited oxidative stress injury, which was in line with the observations reported in a study conducted before (Samad et al., 2023). Each of the administration groups was able to significantly reduce the level of MDA (*p* < 0.05 for all). High dose GLJ and Donepezil showed significantly increase the activity of SOD (*p*<0.05 for all). High dose GLJ and EGB can significantly increase the activity of GSH-Px (*p* < 0.05 for all). Moreover, GLJ exhibited a more comprehensive amelioration of oxidative stress than the two positive drugs, as evidenced by its superior efficacy in upregulating both SOD and GSH-Px activities. In this study, the levels of TNF-α, IL-1β, and IL-6 in the Model group were significantly increased (*p* < 0.05, *p* < 0.05, *p* < 0.01) compared with the Ctrl group, which was similar to the results in the literature studies on D-gal-induced cognitive impairment (Li et al., 2023). High dose GLJ can significantly increase the levels of TNF-α, IL-1β and IL-6 (*p* < 0.05 for all). Besides, EGB showed significant callback effects on IL-1β (*p* < 0.05). In contrast, donepezil did not exert any significant effect on the measured inflammatory cytokines. These results indicate that the regulatory effect of GLJ on the levels of inflammatory cytokines was superior to that of EGB and Donepezil. Overall, these results show that GLJ provided a protective benefit in the rat model of MCI by reducing oxidative stress and inflammation.

**FIGURE 3:**
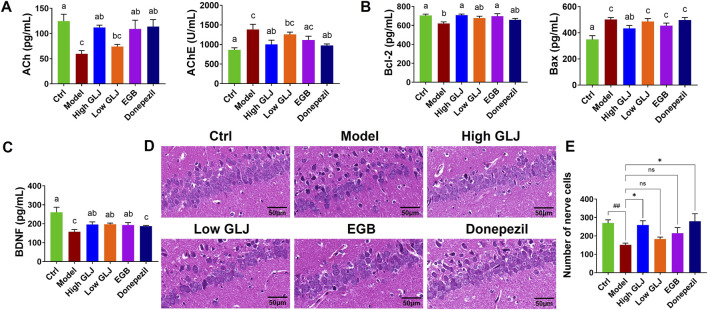
The hippocampal biochemical indices and the histopathological examination of the hippocampal CA1 region of rats in each group. **(A)** The hippocampal levels of cholinergic index in each group of rats. **(B)** The hippocampal levels of apoptotic indices of each group of rats. **(C)** The hippocampal levels of neurotrophic indices of each group of rats. **(D)** Histopathological examination of the hippocampal CA1 region by HE staining (×400). **(E)** Quantitative analysis of neuronal survival in the hippocampal CA1 region. Statistical significance was determined by one-way ANOVA followed by Tukey’s post hoc test. #*p* < 0.05 vs. Ctrl group; *p < 0.05 vs. Model group, ns, not significant. Letters a, b, c indicates groups with *p* < 0.05 by Tukey’s test.

GLJ Improves Hippocampal Cholinergic Function, Cell Survival, and Histoarchitecture in a Rat Model of MCI Analysis of the hippocampal cholinergic index revealed that the levels of ACh decreased significantly, while the levels of AChE increased remarkably in the Model group compared to the Ctrl group (*p* < 0.01 for all; Figure 3A). High dose GLJ, EGB and Donepezil showed significantly increase in the level of ACh (*p* < 0.01, *p* < 0.05, *p* < 0.0). High dose GLJ and Donepezil showed significantly increase the activity of AChE (*p* < 0.05, *p* < 0.01). However, GLJ of low dose has no significant regulatory effect on ACh and AChE. The hippocampal levels of BDNF and Bcl-2 decreased significantly (*p* < 0.01 for all), while the levels of Bax (*p* < 0.01) increased in the rat model of MCI, compared to those of the Ctrl group. High dose GLJ, low dose GLJ and EGB showed significant increase in the level of BDNF (*p* < 0.05 for all; Figures 3B,C) compared with the model group. Donepezil has no significant regulatory effect on BDNF. High dose GLJ showed significant callback effects on Bax (*p* < 0.05). However, none of the other administration groups showed any reduction effect. High dose GLJ and EGB showed significant callback effects on Bcl-2 (*p* < 0.05 for all). Low dose GLJ and Donepezil have no significant regulatory effect on Bcl-2.

The hippocampal CA1 region in MCI rats displayed key pathological features, including enlarged intercellular spaces, nuclear shrinkage in pyramidal cells, and a reduced neuronal count (Figures 3D,E). However, treatment with EGB, Donepezil, and a high dose of GLJ alleviated the damage to the hippocampal neurons. Collectively, the data indicate that GLJ exhibited a dose-dependent efficacy in restoring hippocampal pathology. The high dose restored all aberrant cholinergic, neurotrophic, and apoptotic indicators, while the low dose and positive controls produced a more limited restorative effect.

### 3.3 Effects of GLJ on the serum and hippocampal metabolites

#### 3.3.1 Stability evaluation of LC-MS system

The total ion current chromatograms are depicted in Supplementary Figures S5,S6. To monitor the stability of the LC-MS system, ten ions having different m/z values and retention times were extracted from each QC sample for verifying the validation strategy (Supplementary Tables S2,S3). RSDs value of the retention time were 5.93 × 10^-4^–4.14 × 10^−3^ and 1.71 × 10^-3^–1.04 × 10^−2^ for the serum and hippocampal samples, respectively, the m/z values were 1.60 × 10^-7^–8.95 × 10^−7^, 1.62 × 10^-7^–2.47 × 10^−6^, respectively, and the relative peak areas were 4.67 × 10^-2^–1.11 × 10^−1^ and 1.34 × 10^-2^–9.02 × 10^−2^, respectively. The results show that the instrument has good stability, and the test results are reliable.

#### 3.3.2 Stability evaluation of LC-MS system

**FIGURE 4:**
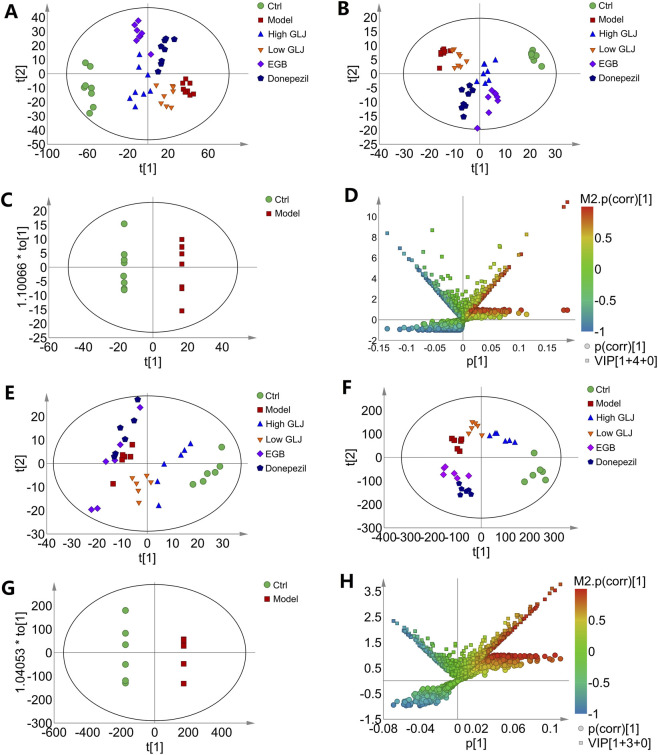
Score plots of metabolomics of the serum and hippocampus samples. **(A)** PCA scores plot of serum samples; **(B)** PLS-DA scores plot of serum samples; **(C)** OPLS-DA scores plot of serum samples; **(D)** V + S-plot of OPLS-DA model C; **(E)** PCA scores plot of hippocampus samples; **(F)** PLS-DA scores plot of hippocampus samples; **(G)** OPLS-DA scores plot of hippocampus samples; **(H)** V + S-plot of OPLS-DA model **(G)**.

The serum and hippocampal metabolite profiles of all groups were reflected by PLS-DA analysis. The scatter plot revealed differences in metabolic profiles between rat groups in both serum and hippocampal samples. There were distinct differences among the serum metabolite profiles of all the groups, as depicted in the PCA score plot (Figure 4A). Subsequent analysis using a supervised PLS-DA model revealed a distinct separation among the scattered data points of the different groups (Figure 4B). Similarly, for hippocampal samples, the overall metabolic profile of the Ctrl and Model group were distributed in two quadrants (Figure 4E), and each administration group was distributed between the Ctrl and Model group (Figure 4F), suggesting that the overall metabolic profile of MCI rats was regulated after administration. The data generated by the PLS-DA model of serum and hippocampal samples were further subjected to 200 random permutation tests, which revealed that the values of R2 and Q2 were closely aligned. The model had a slightly steeper slope, and its vertical axis intercept was below zero. These findings indicated that the model was not overfitted and confirmed the reliability of the results (Supplementary Figure S7).

The differential metabolites in the serum and hippocampal samples of the Ctrl and Model groups were further explored using the OPLS-DA method (Figures 4C,G). This result showed that the serum and hippocampal metabolic profiles underwent significant alterations in the rat model of MCI. The models for the serum (R^2^X = 0.417, R^2^Y = 0.936, Q^2^ = 0.408, and *P* = 5.97 × 10^−4^ for CV-ANOVA validation) and hippocampal samples (positive ion mode: R^2^X = 0.477, R^2^Y = 0.975, Q^2^ = 0.692, and *P* = 6.51 × 10^−3^ for CV-ANOVA validation) exhibited a good fit and accurately predicted the classified data. The potential biomarkers were subsequently screened from the S-plots and VIP values (VIP >1 and *p* < 0.05), as depicted in the V + S-plot (Figures 4D,H). The differential metabolites were then identified by comparing the accurate masses and product ions with related literature and databases. A total of 24 differential serum metabolites (Supplementary Table S4) and 19 differential hippocampal metabolites (Supplementary Table S5) were finally identified as the key markers that distinguished the Model and Ctrl groups.

To elucidate the regulatory effect of GLJ on the serum and hippocampal metabolites of the rat model of MCI, the recovery rates were calculated for the different treatment groups using the following formula: recovery rate = [(relative peak area of the treatment group - relative peak area of Model group)/(relative peak area of Ctrl group - relative peak area of Model group)] * 100% (Supplementary Tables S4,S5). The findings revealed that GLJ exerted the most significant overall regulatory effect on the metabolic profiles when administered at a high dose. A high dose of GLJ significantly restored the profiles of 16 and 15 differential metabolites in the serum and hippocampal tissues, respectively. These differential metabolites included hexadecasphinganine, phytosphingosine, cholic acid, linoleoyl carnitine, deoxycholic acid, 6 forms of lysoPC, eicosapentaenoic acid, linolenic acid (ALA), palmitoleic acid, oleamide, and arachidonic acid (AA) in the serum. In serum samples, the total and average recovery rates for the high-dose GLJ group were 1015.39% and 63.46%, respectively. Furthermore, 12, 13, and 8 metabolites were significantly altered by low-dose GLJ, EGB, and Donepezil, respectively, compared to the Model group. Treatment with a high dose of GLJ significantly altered the hippocampal levels of oleic acid, nicotinamide, cis-aconitic acid, citric acid, valine, aspartic acid, triethylamine, pyroglutamic acid, linoleic acid (LA), norleucine, adenosine, glutamic acid, tryptophan, and ALA, and the total and average recovery rates were 1354.44% and 90.32%, respectively. In the hippocampus, 8 metabolites were adjusted significantly by GLJ of low dose; 9 and 11 metabolites were adjusted significantly by EGB and Donepezil, respectively. The results revealed that GLJ of high dose showed better regulation on the metabolic profile of Model group rats, compared to the two positive drugs, in both serum and hippocampus.

#### 3.3.3 Correlation analysis of the differential metabolites and biochemical indices

**FIGURE 5:**
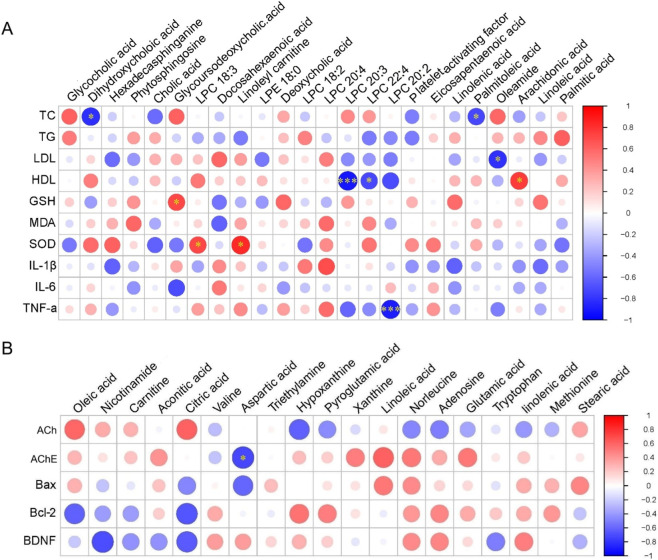
Heatmaps depicting the correlation between the differential metabolites and biochemical indices in the **(A)** serum and **(B)** hippocampal samples. *p < 0.05, **p < 0.01, and ***p < 0.005.

The results of the correlation analyses are depicted in Figure 5. In this context, red represents a positive correlation, while blue indicates a negative correlation. The correlation coefficients (|r|) can vary from 1.0, which signifies the strongest positive correlation, to −1.0, indicating the strongest negative correlation, with a value of 0 representing no correlation at all. |r| > 0.5 and P < 0.05 represents a significant correlation between the two. The results of correlation analysis for the serum samples revealed that the TC levels exhibited a significant negative correlation with (3β,7α)-3,7-Dihydroxychol-5-en-24-oic acid and palmitoleic acid. Similarly, there was a significant negative correlation between LDL-C and oleamide, and between HDL-C and lysophosphatidylcholine (LPC) (20:3 and 22:4). However, there was a significant positive correlation between HDL-C and AA, and between GSH and glycoursodeoxycholic acid. SOD exhibited a significant positive correlation with LPC (18:3) and linoleyl carnitine; and there was a significant negative correlation between TNF-α and LPC (20:2). Additionally, there was a significant negative correlation between AChE and aspartic acid in the hippocampus.

#### 3.3.4 Metabolic pathway analysis

**FIGURE 6:**
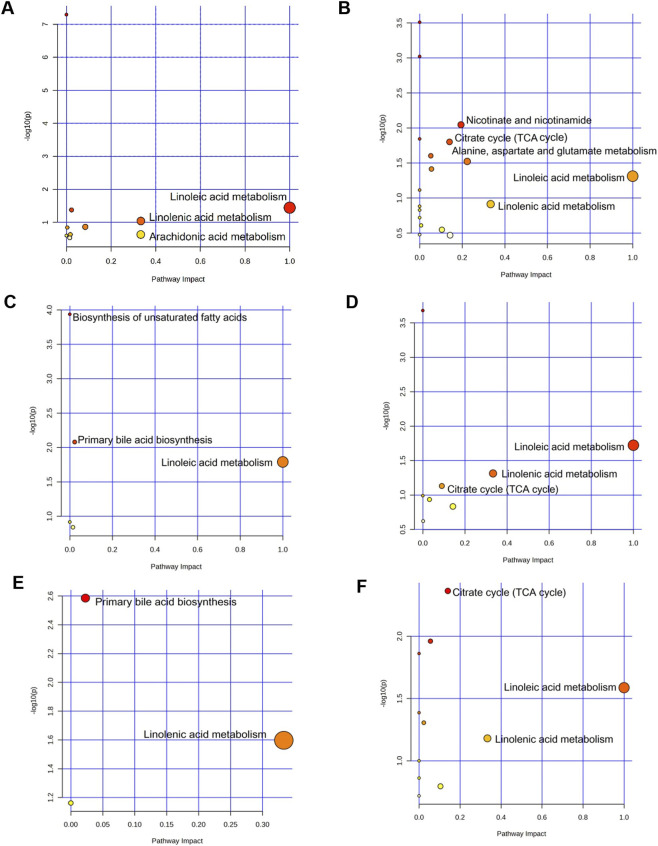
Metabolic pathway analysis of serum and hippocampal samples. **(A)** Pathways associated with GLJ treatment in serum. **(B)** Pathways associated with GLJ treatment in the hippocampus. **(C)** Pathways associated with EGB treatment in serum. **(D)** Pathways associated with EGB treatment in the hippocampus. **(E)** Pathways associated with Donepezil treatment in serum. **(F)** Pathways associated with Donepezil treatment in the hippocampus.

The correlation between the differential metabolites affected by GLJ and those affected by the two positive control drugs, were further investigated by exploring the relevant metabolic pathways using Metaboanalyst 6.0. Pathways with an impact value greater than 0.1 were identified as the primary pathways. Ultimately, the differential metabolites of GLJ were involved in 4 pathways, including linoleic acid metabolism, linolenic acid metabolism and arachidonic acid metabolism (Figure 6A). Those of EGB and Donepezil were associated with only one pathway each: linoleic acid metabolism and linolenic acid metabolism, respectively (Figures 6B,C). In the hippocampus, the metabolites altered by GLJ were implicated in several pathways including the TCA cycle and amino acid metabolism (Figure 6D). EGB and Donepezil also showed involvement in the TCA cycle and fatty acid metabolism pathways (Figures 6E,F). The above results suggest that both GLJ and the two positive drugs ameliorate MCI by modulating lipid metabolism, particularly linoleic acid. Correlation analysis of differential metabolites and biochemical indicators further reveals significant associations between oxidative stress, inflammatory markers, and lipid components. Research indicates Linoleic acid (LA) and α-linolenic acid (ALA) modulate key physiological processes, including apoptosis and inflammatory pathways. Deficiencies in LA and ALA have also been linked to neurological disorders, psychiatric conditions, and metabolic dysregulation (Kim and Song, 2024). Thus, future experiments should investigate whether Guilingji’s regulation of linoleic acid and related lipids influences neuroinflammation, thereby contributing to MCI improvement.

### 3.4 Effect of GLJ on the expression of GPR120, NF-κB and TNF-α in the hippocampus

**FIGURE 7:**
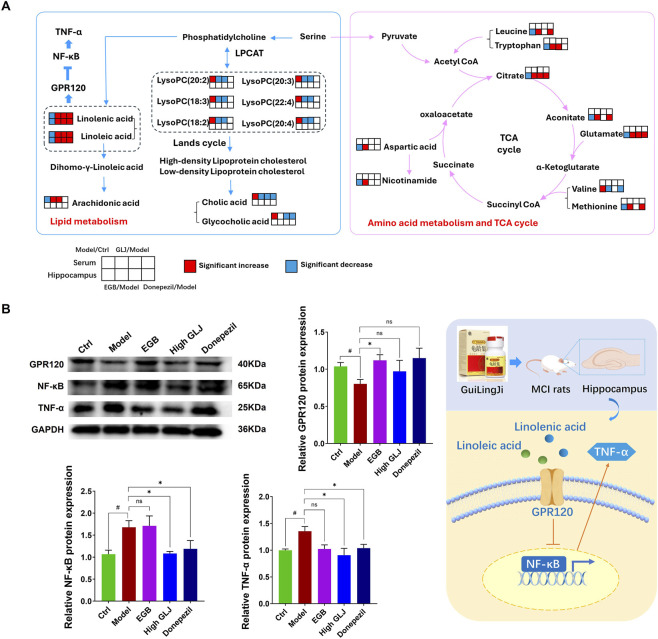
**(A)** Effects of GLJ on the expression of proteins related to neuroinflammation pathway in the hippocampus (n = 3). **(B)** Schematic diagram integrating the metabolic network and the related neuroinflammation pathway associated with the differential metabolites altered by GLJ and the two positive drugs in MCI rats. Statistical significance was determined by one-way ANOVA followed by Tukey’s *post hoc* test. #*p* < 0.05 vs. Ctrl group; *p < 0.05 vs. Model group.

Based on integrated metabolomics of serum and hippocampus with KEGG enrichment analysis, Figure 7A illustrates the metabolic pathways associated with GLJ treatment and its potential mechanism of action against MCI. In this study, significant modulation of LA and ALA levels was observed in both serum and hippocampal tissues, and previous research has shown that LA and ALA exert their biological effects through binding to GPR120 receptors, which subsequently inhibits the NF-κB signaling pathway. This inhibition leads to downregulation of TNF-α expression, resulting in attenuated neuroinflammatory responses and consequent improvement in cognitive dysfunction (Kim and Song, 2024). Therefore, the relative levels of GPR120, NF-κB and TNF-α in the hippocampus were further confirmed. The expression of GPR120 was significantly downregulated in the model group, whereas the levels of NF-κB and TNF-α were markedly elevated. Treatment with GLJ and the two positive drugs significantly modulated the expression of GPR120, NF-κB and TNF-α. Notably, GLJ exhibited a more pronounced regulatory effect compared to the two positive drugs (Figure 7B).

## 4 Discussion

GLJ was originally a revered formulation used by imperial families during the Ming and Qing dynasties to delay aging. Modern clinical and pharmacological studies have demonstrated their efficacy in enhancing cognitive function. However, the therapeutic effects and underlying mechanisms of GLJ in MCI remain unclear. This study employed a D-gal combined with semi-high-fat diet induced MCI animal model. Given that GLJ is a multi-component herbal formula expected to act *via* polypharmacology, we employed a dual-control design: donepezil, a conventional single-target Western drug, and ginkgo biloba tablets, a clinically validated herbal preparation (Pagotto et al., 2024; Yang et al., 2025). This approach allowed for a more comprehensive and comparable evaluation of GLJ’s efficacy within a similar multi-target therapeutic paradigm. Through metabolomics methods, owing to its capacity to holistically assess multi-target synergistic effects, elucidate systemic regulatory networks, and correlate pharmacodynamic phenotypes, offers a superior approach over conventional methods for deciphering the pharmacological mechanisms of traditional Chinese medicine (TCM) compound formulations, the effect and pharmacological mechanism of GLJ in improving the efficacy of MCI were explored.

Animal models of aging induced by D-gal are a popular tool in research and are extensively used to explore the mechanisms underlying aging, which is characterized by elevated levels of free radicals and oxidative stress (Xiao et al., 2024). Meanwhile, substantial evidence indicates that risk factors for cardiovascular disease are associated with cognitive dysfunction, with some major factors further contributing to its decline (Wang et al., 2023). And high-fat diet can cause disorders of lipid metabolism, increase the levels of blood lipids, and induce widespread inflammation. This may adversely affect the brain, which in turn induces oxidative stress that can lead to cognitive dysfunction (Mei et al., 2023; Ooi et al., 2022; Tan and Norhaizan, 2019). Therefore, the MCI rat model was established using D-gal combined with semi-high-fat diet, cognitive-related high-risk cardiovascular factors were introduced to make the model more consistent with clinical pathological change (Li et al., 2019; Peng et al., 2017). In this study, the Morris water maze test confirmed that this combined intervention successfully induced significant memory deficits in the rats, along with substantial increases in blood lipids, oxidative stress, and inflammatory damage, all of which were alleviated by GLJ. Hippocampus is the primary cognitive module. D-gal and a semi-high-fat diet impair hippocampal functions and elevate the risk of cognitive impairment (Samad et al., 2023; Yang et al., 2024). A previous study demonstrated that significant neuronal apoptosis was observed in the hippocampus of AD patients, and a significant decrease of BDNF, Ach levels (Mineur et al., 2022; Yang et al., 2024). This study revealed significant pathological damage in the hippocampus of MCI model rats, accompanied by elevated AChE, reduced ACh and BDNF levels, and increased neuronal apoptosis. GLJ treatment effectively ameliorated these histopathological and biochemical alterations. Comparative analysis indicated that GLJ demonstrated the most potent overall efficacy among the treatments tested, as it positively influenced a broader range of the assessed indicators than the two positive control drugs.

Metabolomic profiling of serum and hippocampal samples from the rat model of MCI, using LC-MS, identified altered concentrations of multiple unsaturated fatty acids. These changes indicated dysfunction in several metabolic pathways associated with lipid metabolism. Previous studies have demonstrated that unsaturated fatty acids are likely involved in preventing and alleviating mental disorders, including dementia, MCI, depression, and others (Gao et al., 2022). The current research showed that there was a significant reduction in the levels of polyunsaturated fatty acids (PUFAs), including LA, ALA, and AA, in the rat model of MCI. The observed therapeutic benefits of GLJ in the multi-factorial MCI model may be partly attributable to the synergistic actions of its key fatty acid constituents, LA and ALA. These essential fatty acids are integral to neuronal membrane structure and serve as precursors for anti-inflammatory and pro-resolving lipid mediators, such as neuroprotectins derived from ALA (Dhillon et al., 2023; Kim and Song, 2024). ALA can decrease the levels of MDA and nitric oxide, increase the content of glutathione in the hippocampal, reduced the expression of various inflammatory factors and prevented neuronal loss in the hippocampal CA1 region (Tofighi et al., 2021). ALA can also inactivate extracellular regulatory protein kinase (ERK) and nuclear factor-κB (NF-κB), by inhibiting the corresponding pathways, to alleviate Aβ-induced neuroinflammation and pathological injury to the brain tissues (Zhu et al., 2021). In the D-gal plus semi-high-fat diet model used here, which is characterized by significant metabolic dysregulation, oxidative stress and systemic inflammation, LA and ALA likely exert their effects by alleviation of systemic and hippocampal oxidative stress, indicated by increased SOD activity and decreased MDA levels, along with the reduction in inflammatory cytokines including TNF-α, IL-1β, and IL-6, aligns with the established antioxidant and anti-inflammatory properties of these fatty acids. Therefore, within the GLJ formulation, LA and ALA are posited to exert their neuroprotective effects by modulating metabolic levels, activating the downstream GPR120 receptor, and thereby indirectly inhibiting the NF-κB pathway. This mechanism underscores their role in mitigating neuroinflammation and cognitive impairment through an indirect, receptor-mediated cascade.

Additionally, the aberrantly elevated serum levels of LPC in the rat model of MCI was also observed in this experiment. Studies have shown that LPC triggers the inflammasome, which then leads to pyroptosis, ultimately worsening neurodegeneration (Zhu et al., 2021). Additionally, LPC can promote the generation of amyloid Aβ1-42 oligomers and aggregation of neurotoxic proteins, which leads to neurotoxicity. Therefore, inhibiting the generation of LPC may serve as an effective intervention strategy for neurodegenerative diseases (Law et al., 2019). The present study revealed that GLJ could improve the cognitive function in the MCI rats by adjusting the serum levels of LPC.

The results of hippocampal metabolomics indicated that the level of glutamate and aspartate present a significant decrease in MCI rats. It has been reported that imbalances in glutamate and aspartate metabolism are associated with various neurodegenerative diseases (Andersen et al., 2021a), cognitive impairment is often accompanied by reduced levels of glutamine and aspartate in the brain (Andersen et al., 2021a; Andersen et al., 2021b; Singh et al., 2020). In this study, GLJ can improve brain function and cognition by upregulating the levels of glutamate and aspartate in hippocampal of MCI rats. Furthermore, the hippocampal levels of Nicotinamide (NAM) decreased in the rat model of MCI. NAM is a crucial building block for nicotinamide adenine dinucleotide (NAD+), and it is generated through the NAD + salvage pathway. The levels of NAD directly affect the glycolytic tricarboxylic acid (TCA) cycle and mitochondrial oxidative phosphorylation. Previous studies have demonstrated that NAM can mitigate the deposition of Aβ plaques and hyperphosphorylation of tau protein, increase mitochondrial resistance to oxidative stress, enhance autophagy-lysosome processes, activate the signal transduction pathways for neuronal survival and synaptic plasticity, and mitigate neuronal dysfunction to alleviate cognitive deficits in AD (Hao et al., 2022; Villeda-González et al., 2020). GLJ may alleviate MCI by regulating the levels of NAM to protect neuronal function. The research additionally disclosed a marked reduction in the hippocampal levels of citric acid and its downstream, aconitic acid, within the MCI rat model. The contents of various essential amino acids involved in the TCA cycle, including aspartic acid, leucine, glutamic acid, pyroglutamic acid, tryptophan, and methionine, also decreased significantly. Previous studies have reported that the levels of citric acid, succinic acid, and fumaric acid in the hippocampal TCA cycle are significantly lower in diabetic rats with cognitive dysfunction, suggesting that the impairment of cognitive function could be attributed to a reduction in energy metabolism (Campbell, 2022; Gao et al., 2019). The present study demonstrated that GLJ could improve cognitive function of the rat model of MCI by regulating amino acid and energy metabolism.

We emphasize that GLJ, as a complex formulation, contains multiple bioactive components capable of engaging with various molecular targets. It is noteworthy that the proposed involvement of the GPR120/NF-κB pathway in this study differs from mechanisms previously reported for GLJ in other cognitive impairment models, such as the reduction of amyloid-β plaque burden in APP/PS1 transgenic mice (Ren et al., 2022). The APP/PS1 model primarily interrogates Aβ-centric pathology, whereas the D-gal combined with semi-high-fat diet induced MCI model, focuses on the metabolic dysregulation and systemic inflammation. Therefore, the anti-inflammatory and metabolic-modulating effects (potentially *via* GPR120/NF-κB and related pathways) likely come to the forefront as the dominant observable mechanism. While this study provides evidence supporting the potential of GLJ in ameliorating multi-factorial MCI, several limitations should be acknowledged. The mechanistic link between GLJ and the proposed GPR120/NF-κB pathway remains circumstantial. Although GLJ administration downregulated NF-κB signaling and its downstream inflammatory mediators, which is consistent with GPR120 activation, we did not perform direct loss-of-function or gain-of-function experiments targeting GPR120 itself. Therefore, the specific contribution of GPR120 to the observed benefits requires further validation.

## 5 Conclusion

The onset of MCI is mediated by complex pathological mechanisms, including oxidative stress, inflammatory responses, neuronal damage in the hippocampus, and metabolic disorders. The present study demonstrated that treatment with GLJ alleviated MCI by modulating the levels of oxidative stress, the inflammatory response, cholinergic function, neuronal apoptosis, and neurotrophy, and significantly improved the disturbance in amino acid and fatty acid metabolism. Furthermore, the treatment specifically modulates LA and ALA levels, activates their cognate receptor GPR120, and consequently suppresses NF-κB signaling and downstream TNF-α expression. This mechanism effectively attenuates neuroinflammation, thereby ameliorating MCI. The benefits of GLJ in treating MCI stem from its ability to act on multiple targets simultaneously. However, the intricate mechanisms underlying the therapeutic effects of GLJ on MCI necessitate further exploration and validation. The present study serves as a guide for the development of therapeutic strategies for MCI in future.

## Data Availability

The original contributions presented in the study are included in the article/Supplementary Material, further inquiries can be directed to the corresponding authors.
