# Supplementary material for: GuiLingJi ameliorates mild cognitive impairment by targeting unsaturated fatty acid metabolism to inhibit GPR120/NF-κB mediated neuroinflammation
Source: Front Pharmacol. 2026 Mar 11;17:1729885. doi: 10.3389/fphar.2026.1729885 (PMC13013273; doi:10.3389/fphar.2026.1729885)
Supplement: Supplementary file 1 [file Supplementaryfile1.docx]

Supplementary Material

**1. Composition and botanical authentication of the GLJ formulation**

**Supplementary Table 1** Composition and botanical authentication of the GLJ formulation

| Botanical Name | Family | Official Medicinal Part Name |
| --- | --- | --- |
| *Panax ginseng* C.A.Mey. | Araliaceae | Ginseng Radix et Rhizoma |
| *Lycium chinense* Mill. | Solanaceae | Lycii Fructus |
| *Syzygium aromaticum* (L.) Merr. & L.M.Perry | Myrtaceae | Caryophylli Flos |
| *Cyathula officinalis* K.C.Kuan | Amaranthaceae | Cyathulae Radix |
| *Cynomorium coccineum* L. | Cynomoriaceae | Cynomorii Herba |
| *Rehmannia glutinosa* (Gaertn.) DC. | Orobanchaceae | Rehmanniae Radix |
| *Cullen corylifolium* (L.) Medik. | Fabaceae | Psoraleae Fructus |
| *Cuscuta chinensis* Lam. | Convolvulaceae | Cuscutae Semen |
| *Eucommia ulmoides* Oliv. | Eucommiaceae | Eucommiae Cortex |
| *Cistanche deserticola* Ma | Orobanchaceae | Cistanches Herba |
| *Glycyrrhiza glabra* L. | Fabaceae | Glycyrrhizae Radix et Rhizoma |
| *Asparagus cochinchinensis* (Lour.) Merr. | Asparagaceae | Asparagi Radix |
| *Epimedium sagittatum* (Siebold & Zucc.) Maxim. | Berberidaceae | Epimedii Herba |
| *Amomum uliginosum* J.Koenig | Zingiberaceae | Amomi Fructus |

**2. Three orthogonal fingerprinting analyses for the GLJ extract**

**2.1 UPLC fingerprint of GLJ**

2.1.1 Sample preparation

An accurately weighed 1.00 g sample of GLJ powder was ultrasonically extracted with 50 mL of methanol at room temperature for 30 min. The extracts were filtered and concentrated to be dried. The residue was dissolved in LC-MS grade methanol, and the solution was completely transferred into a 2 mL volumetric flask. The solutions were filtered through a nylon syringe filter (0.22 mm) for UHPLC-MS analysis.

2.1.2 Chromatographic condition

Waters ACQUITY UPLC HSS T3 (2.1×100 mm；1.8 μm) was used with acetonitrile and water in gradient elution mode. The detective wavelength was 203 nm, the flow rate was 0.3 mL/min, and the column temperature was 30 ℃. Twenty batches of GLJ were analyzed. The superimposed Ultra-Performance Liquid Chromatography **(**UPLC) fingerprint of 20 batches of GLJ are shown in **Supplementary Figure 1**.

2.1.3 Conclusion

The fingerprints of GLJ were established with 19 common peaks by UPLC-DAD, the similarity between the 20 batches of GLJ and their reference spectra was greater than 0. 932，the similarity between 20 batches was greater than 0. 820. The results showed that the uniformity of GLJ products is good and the production process is stable.


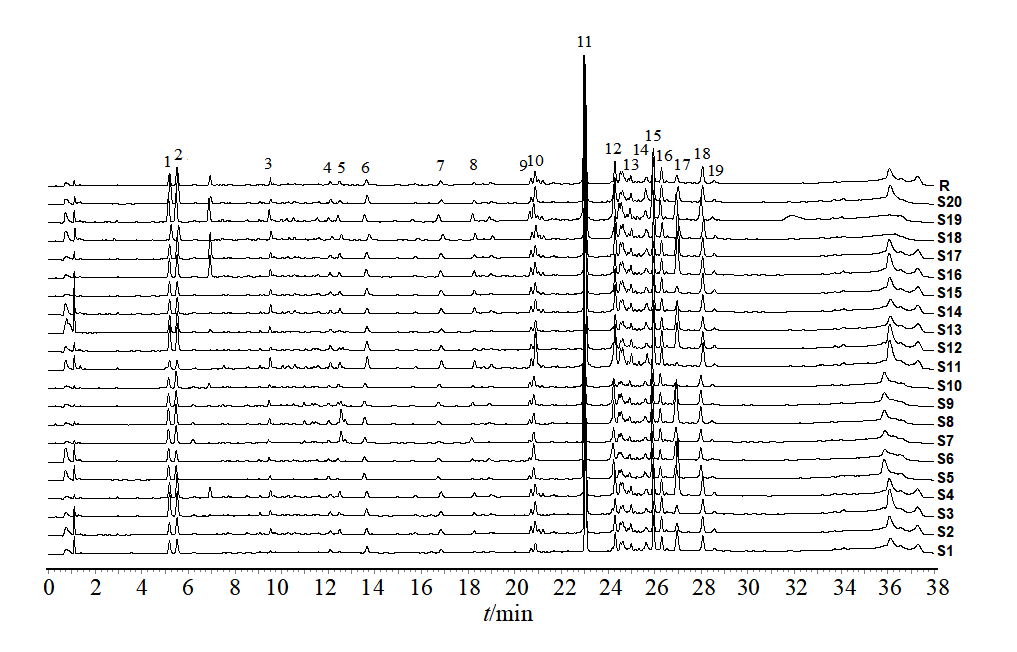


**Supplementary Figure 1** UPLC fingerprint of 20 batches of GLJ

**2.2 GC-MS fingerprint of GLJ**

2.2.1 Sample preparation and GC-MS chromatographic conditions

Accurately weigh 1.0 g of the GLJ powder into a 20 mL headspace vial. The sample was then subjected to headspace solid-phase microextraction (HS-SPME) using a manual injection device equipped with a 65 μm PDMS/DVB fiber. The fiber was exposed to the vial headspace at 60 °C for 40 min after a 20 min equilibration at the same temperature. Following extraction, the fiber was immediately inserted into the GC injection port for thermal desorption for 5 min, which triggered the start of the chromatographic program. The superimposed GC-MS fingerprint of 10 batches of GLJ are shown in **Supplementary Figure 2**.

2.2.2 Conclusion

Headspace solid-phase microextraction coupled with gas chromatography-mass spectrometry (HS-SPME-GC-MS) was used to establish a GC - MS fingerprint of volatile components in GLJ. The Similarity Evaluation System for Chromatographic Fingerprint of Traditional Chinese Medicine （Version 2004A） software was used to identify common peaks. According to the standard mass spectrometry database of the National Institute of Standards and Technology （NIST） and literature search，the names of components were determined，and the peak attribution was identified. There were 68 common peaks in 10 batches of samples, the similarity of all the samples ranged from 0.80~0.95.


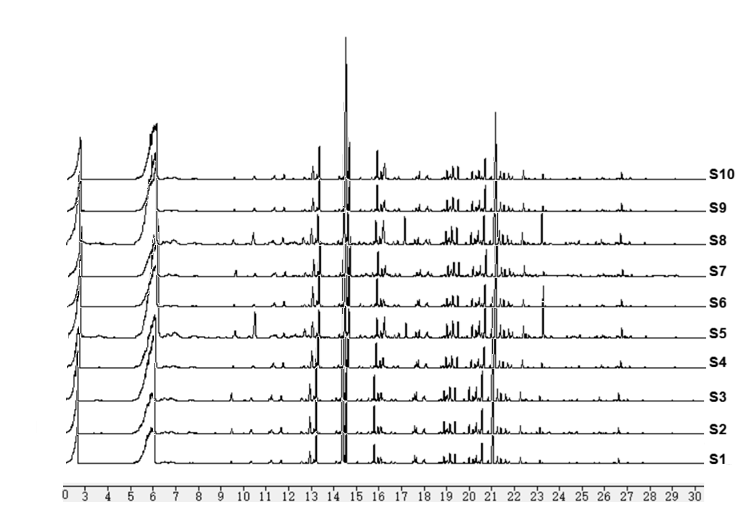


**Supplementary Figure 2** GC-MS fingerprint of 10 batches of GLJ

**2.3 Inorganic elemental fingerprint of GLJ**

2.3.1 Sample preparation

Accurately weighed portions (~0.2 g) of the GLJ powder were digested with 6 mL of concentrated nitric acid in sealed PTFE vessels using a microwave digestion system. After cooling below 70 °C, the digests were dissolved with purified water and diluted to 100 mL in volumetric flasks to prepare the analytical solutions. The inorganic elemental fingerprint of 10 batches of GLJ are shown in **Supplementary Figure 3**.

2.3.2 Conclusion

To evaluate the consistency, reference fingerprint profiles were generated using both the mean and median methods. The cosine similarity between the inorganic element fingerprints of ten GLJ batches and these reference profiles was calculated using IBM SPSS Statistics 22.0. All cosine similarity values exceeded 0.970, indicating a high degree of similarity among the fingerprints across different batches of GLJ.


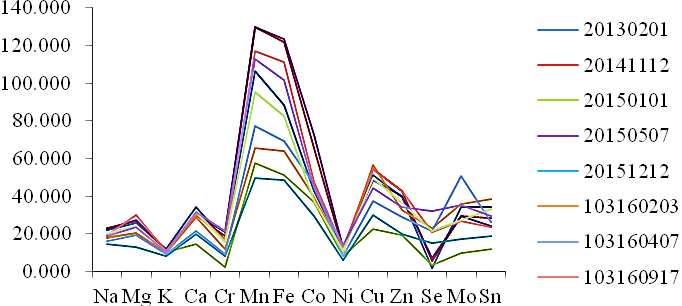


**Supplementary Figure 3.** Inorganic elemental fingerprint of 10 batches of GLJ

**3. Characterization of multiple chemical components of GLJ by UHPLC-MS**

**
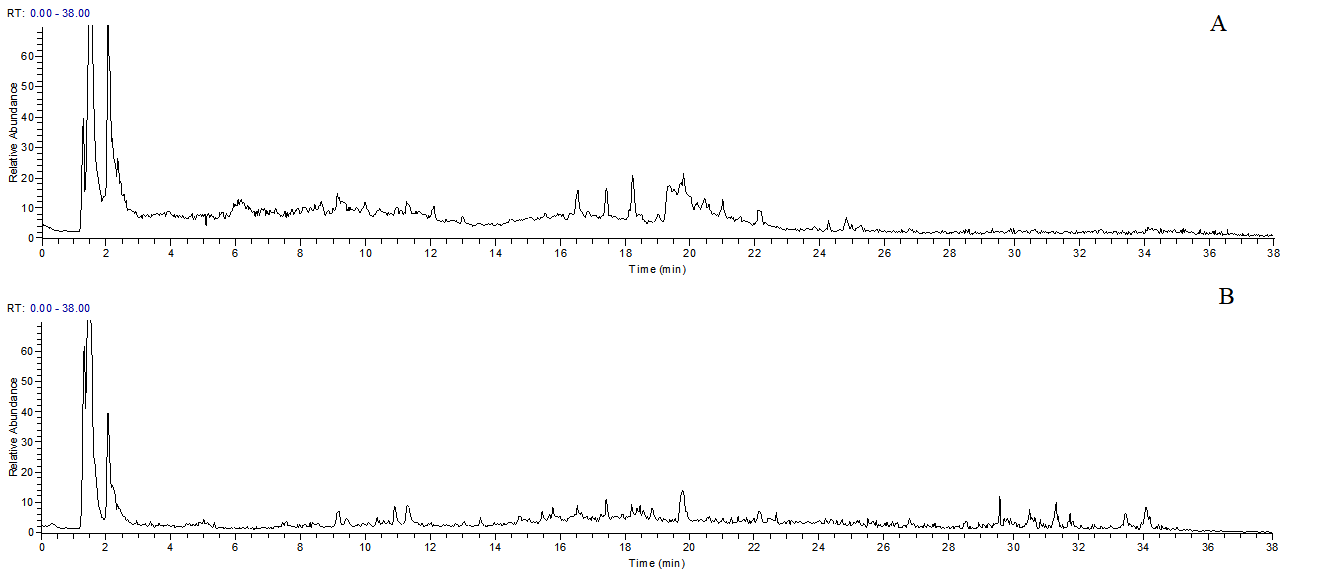
**

**Supplementary Figure 4.** The total ion chromatograms of GLJ in positive (A) and negative (B) ion modes

**4. LC-MS-based metabolomic analysis of serum and hippocampal samples**


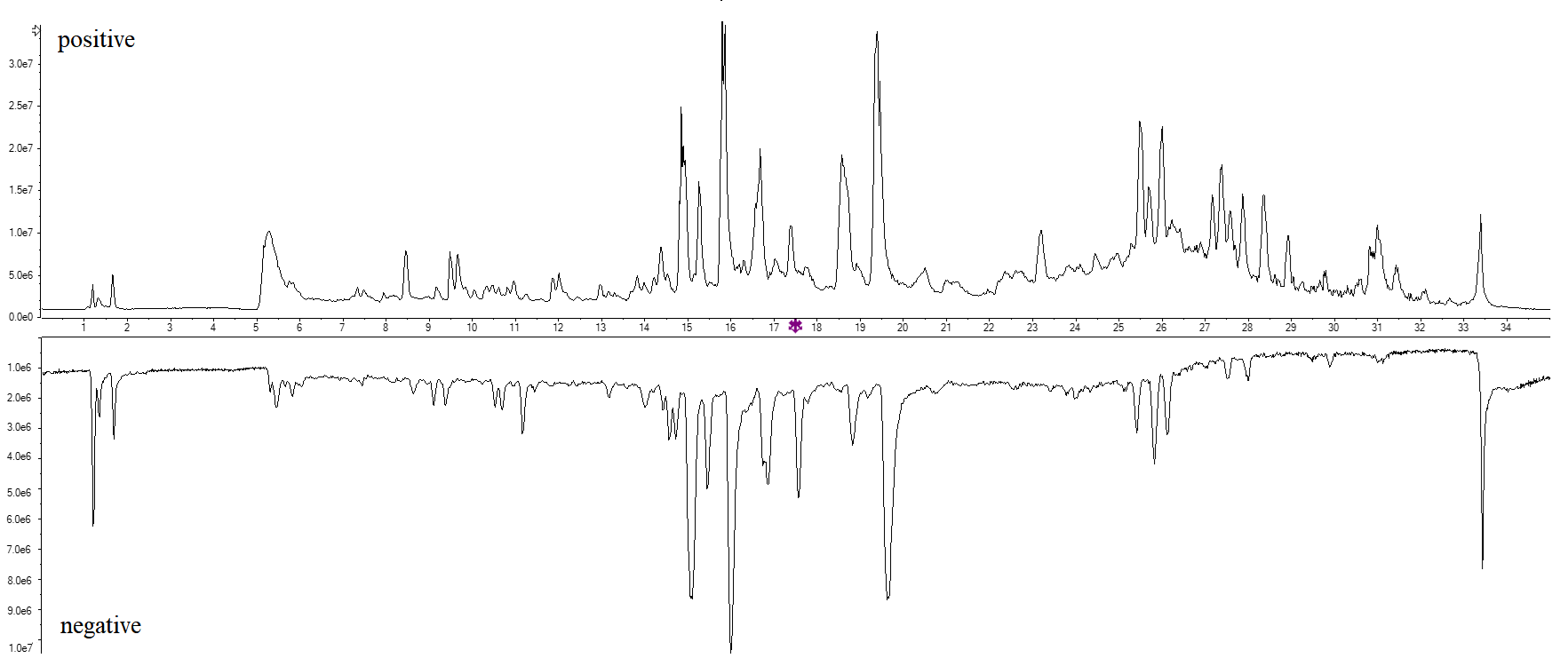


**Supplementary Figure 5.** The total ion chromatograms serum samples of rats in positive and negative ion mode

**Supplementary Table 2** UHPLC-TOF-MS stability monitoring results of serum QC samples

| No. | *t*_R_(min) | RSD (%) of retention time | m/z | RSD (%) of m/z | RSD (%) of relative peak areas | ion |
| --- | --- | --- | --- | --- | --- | --- |
| 1 | 9.09 | 1.73×10^-3^ | 274.2738 | 5.49×10^-7^ | 5.00×10^-2^ | [M+H]^+^ |
| 2 | 10.81 | 1.68×10^-3^ | 407.2797 | 6.15×10^-7^ | 1.11×10^-1^ | [M-H]^-^ |
| 3 | 14.66 | 1.07×10^-3^ | 391.2851 | 8.95×10^-7^ | 4.67×10^-2^ | [M-H]^-^ |
| 4 | 14.74 | 2.02×10^-3^ | 520.3389 | 4.33×10^-7^ | 6.85×10^-2^ | [M+H]^+^ |
| 5 | 17.42 | 4.14×10^-3^ | 548.3711 | 4.41×10^-5^ | 4.85×10^-2^ | [M+H]^+^ |
| 6 | 19.39 | 2.90×10^-3^ | 524.3698 | 4.08×10^-7^ | 7.25×10^-2^ | [M+H]^+^ |
| 7 | 24.07 | 1.43×10^-3^ | 277.217 | 1.60×10^-5^ | 5.61×10^-2^ | [M-H]^-^ |
| 8 | 25.89 | 8.84×10^-4^ | 281.2719 | 1.51×10^-5^ | 6.28×10^-2^ | [M+H]^+^ |
| 9 | 26.18 | 8.20×10^-4^ | 279.2329 | 2.95×10^-7^ | 7.29×10^-2^ | [M-H]^-^ |
| 10 | 27.66 | 5.93×10^-4^ | 255.2335 | 1.60×10^-7^ | 1.09×10^-1^ | [M-H]^-^ |


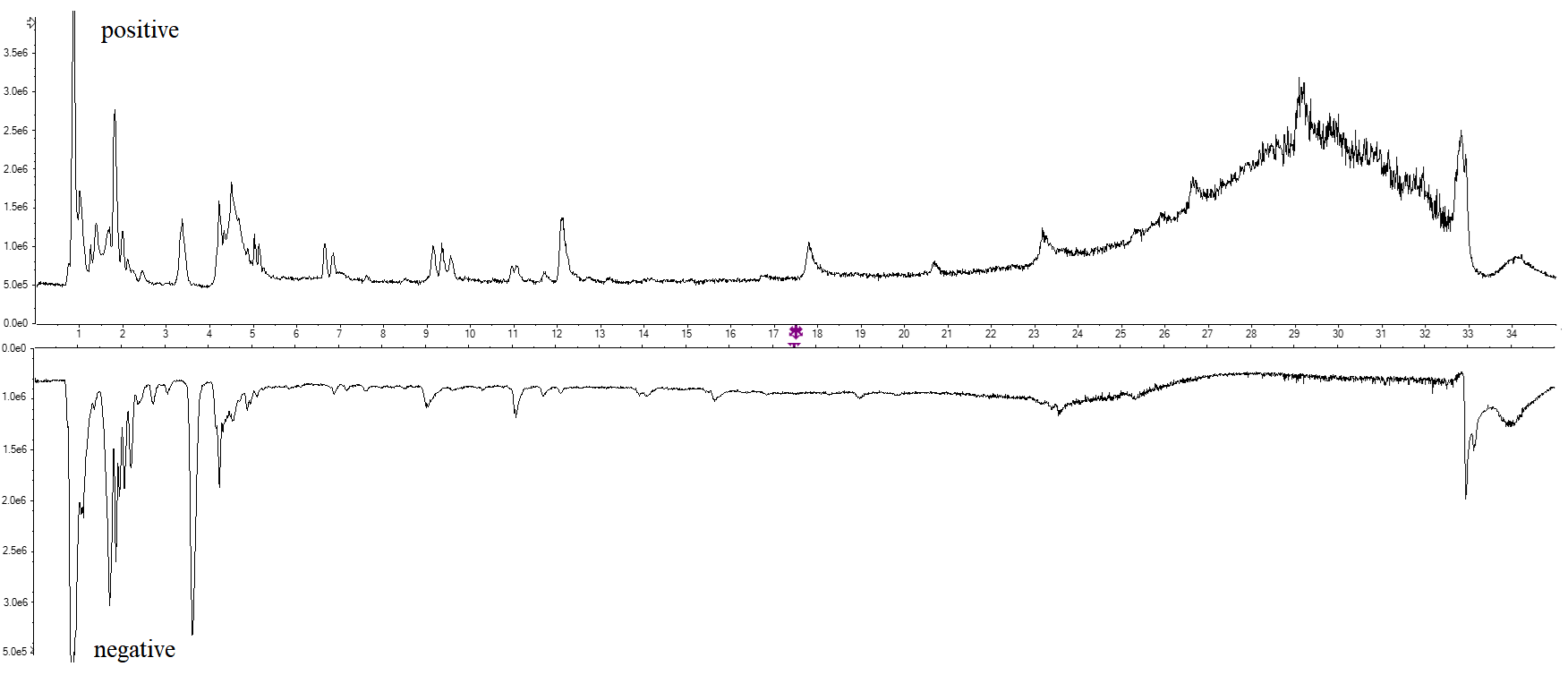


**Supplementary Figure 6** TIC diagram of hippocampus samples of rats in positive (A) and negative (B) ion mode

**Supplementary Table 3** UHPLC-TOF-MS stability monitoring results of hippocampus QC samples

| No. | *t*_R_(min) | RSD (%) of retention time | m/z | RSD (%) of m/z | RSD (%) of relative peak areas | ion |
| --- | --- | --- | --- | --- | --- | --- |
| 1 | 1.11 | 3.70×10^-3^ | 173.0095 | 6.06×10^-7^ | 7.63×10^-2^ | [M-H]^-^ |
| 2 | 1.85 | 1.79×10^-3^ | 135.0312 | 1.08×10^-6^ | 8.36×10^-2^ | [M-H]^-^ |
| 3 | 2.03 | 3.48×10^-3^ | 152.0288 | 2.47×10^-6^ | 7.63×10^-2^ | [M-H]^-^ |
| 4 | 3.49 | 6.18×10^-3^ | 267.0730 | 1.96×10^-6^ | 7.63×10^-2^ | [M-H]^-^ |
| 5 | 4.47 | 1.91×10^-3^ | 130.1585 | 9.31×10^-7^ | 9.02×10^-2^ | [M+H]^+^ |
| 6 | 17.80 | 1.71×10^-3^ | 150.0259 | 1.96×10^-6^ | 2.21×10^-2^ | [M+H]^+^ |
| 7 | 23.04 | 1.04×10^-2^ | 282.2788 | 1.14×10^-6^ | 1.75×10^-2^ | [M+H]^+^ |
| 8 | 26.45 | 7.75×10^-3^ | 284.2944 | 3.46×10^-7^ | 3.20×10^-2^ | [M+H]^+^ |
| 9 | 29.06 | 8.31×10^-3^ | 338.3415 | 1.62×10^-7^ | 1.34×10^-2^ | [M+H]^+^ |
| 10 | 32.78 | 2.36×10^-3^ | 768.7061 | 6.52×10^-7^ | 5.79×10^-2^ | [M+H]^+^ |

A

B

**Supplementary Figure 7** Permutation tests of PLS-DA model of serum (A) and hippocampus (B) samples


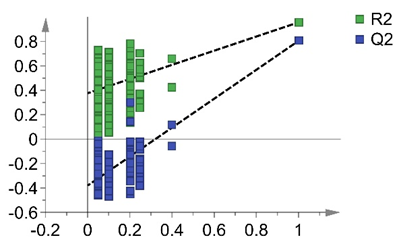

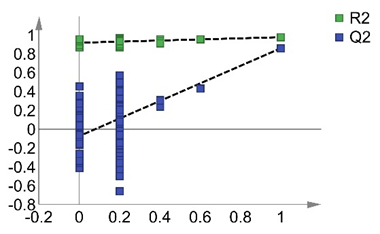


**Supplementary Table 4** The differential metabolites in serum of rat identified by LC-MS

| No. | Name | tR（min） | m/z | ion | Model VS Ctrl | Recovery rate (%) | | | |
| --- | --- | --- | --- | --- | --- | --- | --- | --- | --- |
|  |  |  |  |  |  | High GLJ | Low GLJ | EGB | Donepezil |
| 1 | Glycocholic acid | 8.81 | 464.3017 | [M-H]^-^ | ↑^***^ |  |  | 55.81 | 41.91 |
| 2 | (3β,7α)-3,7-Dihydroxychol-5-en-24-oic acid | 9.05 | 391.2846 | [M+H]^+^ | ↑^***^ |  |  |  |  |
| 3 | Hexadecasphinganine | 9.09 | 274.2738 | [M+H]^+^ | ↑^***^ | 55.64 | 66.50 | 35.58 | 35.33 |
| 4 | Phytosphingosine | 9.26 | 318.3002 | [M+H]^+^ | ↑^***^ | 45.93 | 63.99 |  |  |
| 5 | Cholic acid | 10.81 | 407.2797 | [M-H]^-^ | ↑^*^ | 91.57 |  | 115.69 | 108.61 |
| 6 | Glycoursodeoxycholic acid | 11.65 | 448.3063 | [M-H]^-^ | ↑^*^ |  |  |  |  |
| 7 | LPC (18:3) | 13.52 | 518.3244 | [M+H]^+^ | ↑^***^ | 84.11 | 48.51 | 87.08 |  |
| 8 | Docosahexaenoic acid | 13.66 | 357.2791 | [M+H]^+^ | ↑^*^ |  |  |  |  |
| 9 | Linoleoyl carnitine | 14.15 | 424.3417 | [M+H]^+^ | ↓^***^ | 88.07 | 46.35 | 95.16 | 23.09 |
| 10 | LPE (18:0) | 14.25 | 482.3244 | [M+H]^+^ | ↓^***^ |  |  |  |  |
| 11 | Deoxycholic acid | 14.66 | 391.2851 | [M-H]^-^ | ↑^*^ | 94.64 |  | 96.46 | 84.86 |
| 12 | LPC (18:2) | 14.74 | 520.3389 | [M+H]^+^ | ↑^***^ | 56.45 | 23.39 | 72.71 |  |
| 13 | LPC (20:4) | 14.83 | 544.3385 | [M+H]^+^ | ↑^***^ | 68.16 | 34.78 | 75.65 | 24.94 |
| 14 | LPC (20:3) | 15.84 | 546.3553 | [M+H]^+^ | ↑^***^ | 65.87 |  | 74.37 |  |
| 15 | LPC (22:4) | 17.01 | 572.3706 | [M+H]^+^ | ↑^***^ | 79.77 | 48.61 | 84.18 |  |
| 16 | LPC (20:2) | 17.42 | 548.3711 | [M+H]^+^ | ↑^***^ | 54.96 |  | 86.89 |  |
| 17 | Platelet-activating factor | 19.39 | 524.3698 | [M+H]^+^ | ↑^**^ |  |  |  | 65.49 |
| 18 | Eicosapentaenoic acid | 24.06 | 301.2173 | [M-H]^-^ | ↓^***^ | 38.93 | 44.16 |  | 21.64 |
| 19 | Linolenic acid | 24.07 | 277.2170 | [M-H]^-^ | ↓^***^ | 40.23 | 58.49 | 21 |  |
| 20 | Palmitoleic acid | 25.17 | 253.2172 | [M-H]^-^ | ↓^***^ | 19.19 | 31.63 |  |  |
| 21 | Oleamide | 25.89 | 281.2719 | [M+H]^+^ | ↑^***^ | 65.09 |  | 94.57 |  |
| 22 | Arachidonic acid | 26.03 | 303.2331 | [M-H]^-^ | ↓^***^ | 66.77 | 73.64 | 20.19 |  |
| 23 | Linoleic acid | 26.18 | 279.2329 | [M-H]^-^ | ↓^***^ |  | 64.92 |  |  |
| 24 | Palmitic acid | 27.66 | 255.2335 | [M-H]^-^ | ↓^*^ |  |  |  |  |
| Average Efficacy Index | | | | | | 63.46 | 50.41 | 76.49 | 50.73 |
| The sum of the Efficacy Index | | | | | | 1015.39 | 604.97 | 994.32 | 405.86 |

Note: ↑: up-regulated metabolite ↓: down-regulated metabolite; ^*^*p* < 0.05, ^**^*p* < 0.01, ^***^*p* < 0.005

**Supplementary Table 5** The differential metabolites in hippocampus of rat identified by LC-MS

| NO. | Name | tR（min） | m/z | ion | Model VS Ctrl | Recovery rate (%) | | | |
| --- | --- | --- | --- | --- | --- | --- | --- | --- | --- |
|  |  |  |  |  |  | High GLJ | Low GLJ | EGB | Donepezil |
| 1 | Oleic acid | 0.83 | 280.9078 | [M-H]^-^ | ↑^***^ | 76.31 |  | 180.30 |  |
| 2 | Nicotinamide | 0.85 | 122.9241 | [M+H]^+^ | ↓^*^ | 70.85 |  |  |  |
| 3 | Carnitine | 0.97 | 162.1118 | [M+H]^+^ | ↓^**^ |  |  |  |  |
| 4 | Aconitic acid | 1.11 | 173.0095 | [M-H]^-^ | ↓^***^ | 131.42 |  |  | 99.80 |
| 5 | Citric acid | 1.12 | 191.0193 | [M-H]^-^ | ↓^***^ | 226.12 | 137.33 | 70.80 | 145.19 |
| 6 | Valine | 1.27 | 118.0858 | [M+H]^+^ | ↑^***^ | 111.62 | 70.00 |  | 13.45 |
| 7 | Aspartic acid | 1.65 | 134.0443 | [M+H]^+^ | ↓^*^ | 92.88 |  |  |  |
| 8 | Triethylamine | 1.78 | 102.1278 | [M+H]^+^ | ↓^**^ | 91.05 |  | 150.93 |  |
| 9 | Hypoxanthine | 1.84 | 135.0313 | [M-H]^-^ | ↑^*^ |  |  |  | 48.75 |
| 10 | Pyroglutamic acid | 1.92 | 128.0355 | [M-H]^-^ | ↑^*^ | 151.55 |  |  |  |
| 11 | Xanthine | 2.03 | 151.0261 | [M-H]^-^ | ↑^*^ |  |  |  |  |
| 12 | Linoleic acid | 2.17 | 279.0383 | [M-H]^-^ | ↓^***^ | 89.29 |  | 75.93 | 64.78 |
| 13 | Leucine | 2.46 | 118.0855 | [M+H]^+^ | ↓^***^ | 52.83 | 27.52 |  | 9.95 |
| 14 | Adenosine | 3.49 | 267.0730 | [M-H]^-^ | ↓^***^ | 111.65 |  | 108.70 | 63.77 |
| 15 | Glutamic acid | 4.47 | 130.1585 | [M+H-H_2_O]^+^ | ↓^***^ | 51.62 | 41.16 | 17.01 | 1.06 |
| 16 | Tryptophan | 17.79 | 205.0853 | [M+H]^+^ | ↓^***^ | 50.52 | 35.24 | 27.55 |  |
| 17 | Linolenic acid | 17.80 | 279.1588 | [M+H]^+^ | ↓^***^ | 47.12 | 36.08 | 36.76 | 24.67 |
| 18 | Methionine | 17.80 | 150.0259 | [M+H]^+^ | ↓^***^ |  | 53.43 |  | 16.46 |
| 19 | Stearic acid | 26.45 | 285.2977 | [M+H]^+^ | ↓^***^ |  |  |  |  |
| Average Efficacy Index | | | | | | 90.32 | 50.10 | 74.22 | 44.35 |
| The sum of the Efficacy Index | | | | | | 1354.83 | 400.77 | 667.97 | 487.89 |

Note: ↑: up-regulated metabolite ↓: down-regulated metabolite; ^*^*p* < 0.05, ^**^*p* <0.01, ^***^*p* <0.005
